# Neurodevelopmental Outcomes of Preschoolers with Antenatal Zika Virus Exposure Born in the United States

**DOI:** 10.3390/pathogens13070542

**Published:** 2024-06-27

**Authors:** Sarah B. Mulkey, Elizabeth Corn, Meagan E. Williams, Emily Ansusinha, Robert H. Podolsky, Margarita Arroyave-Wessel, Gilbert Vezina, Colleen Peyton, Michael E. Msall, Roberta L. DeBiasi

**Affiliations:** 1 Zickler Family Prenatal Pediatrics Institute, Children’s National Hospital, Washington, DC 20010, USA; 2 Department of Neurology, The George Washington University School of Medicine and Health Sciences, Washington, DC 20037, USA; 3 Department of Pediatrics, The George Washington University School of Medicine and Health Sciences, Washington, DC 20037, USA; rdebiasi@childrensnational.org; 4 Division of Pediatric Infectious Diseases, Children’s National Hospital, Washington, DC 20010, USA; eansusinha@childrensnational.org; 5 Division of Biostatistics and Study Methodology, Children’s National Hospital, Washington, DC 20010, USA; 6 Division of Radiology, Children’s National Hospital, Washington, DC 20010, USA; 7 Department of Physical Therapy and Human Movement Sciences, Northwestern University Feinberg School of Medicine, Chicago, IL 60611, USA; colleen.peyton1@northwestern.edu; 8 Kennedy Research Center on Intellectual and Neurodevelopmental Disabilities, University of Chicago Medicine, Chicago, IL 60637, USA; mmsall@bsd.uchicago.edu

**Keywords:** infectious disease, child development, executive function, motor, school readiness, congenital infection, congenital Zika syndrome, autism spectrum disorder, developmental delay

## Abstract

Neurodevelopmental outcomes for preschool-age children in the United States with in utero Zika virus (ZIKV) exposure have not yet been reported. We performed a case-control study to assess whether children exposed in utero to ZIKV have abnormal neurodevelopment at age 4–5 years compared to unexposed controls. Thirteen ZIKV-exposed cases that did not have microcephaly or other specific features of congenital Zika syndrome and 12 controls were evaluated between ages 4–5 years. Child neurodevelopment was assessed using the Pediatric Evaluation of Disability Inventory, Behavior Rating Inventory of Executive Function, Peabody Picture Vocabulary Test, Bracken School Readiness Assessment (BSRA), and Movement Assessment Battery for Children (MABC). Caregivers answered questions on the child’s medical history and family demographics. Cases and controls were evaluated at mean (SD) ages 4.9 (0.3) and 4.8 (0.4) years, respectively. Caregivers reported more behavior and mood problems in cases than controls. MABC scores showed more gross and fine motor coordination difficulties among cases than controls. Controls trended towards higher performance on concepts underlying school readiness on BSRA. Three cases had a diagnosis of autism spectrum disorder or global developmental delay. Continued follow-up through school age for children with prenatal ZIKV exposure is needed to understand the impact of in utero ZIKV exposure on motor coordination, cognition, executive function, and academic achievement.

## 1. Introduction

Since the recognition of Zika virus (ZIKV) as a human teratogen amid the 2015–2016 viral outbreak, ongoing research has sought to assess the range and magnitude of neonatal, infant, and child neurodevelopmental outcomes following in utero ZIKV exposure. Congenital disabilities, including microcephaly and other congenital disabilities termed “congenital Zika syndrome” (CZS), are seen in a relatively small proportion of infants exposed to ZIKV in utero, and these children typically have multiple neurological impairments and global developmental delays [1,2,3]. While the majority of infants exposed to ZIKV in utero do not have CZS, microcephaly at birth, or other ZIKV-related congenital disabilities, longitudinal follow-up is recommended for all children with antenatal ZIKV exposure as it is unknown whether exposure to ZIKV during fetal development has long-term impacts on child health, development, growth, and learning and the neurodevelopmental domains that may be most impacted [4,5,6]. Studies assessing early child neurodevelopment in normocephalic children with antenatal ZIKV exposure found indications of lower neurodevelopmental scores for age in cognitive, motor, and language domains [6,7].

Less has been reported about the neurodevelopmental impact of antenatal ZIKV exposure for children in the United States (US) compared to other countries of the Caribbean, Central America, and South America. During the ZIKV epidemic, mosquito-borne ZIKV cases acquired in the continental US were fortunately limited to certain regions of Texas and Florida [6]. The US territories, including Puerto Rico and the US Virgin Islands, had larger numbers of locally acquired ZIKV cases [8]. However, the threat and burden of ZIKV infection during pregnancy for many people in the US was through personal travel to geographic areas where ZIKV was circulating or their sexual partners’ travel to ZIKV-endemic areas, and by emigration to the US from areas of high transmission at the time of conception and early pregnancy [6]. In response to the growing threat of ZIKV and its teratogenic effects in pregnancy, the multidisciplinary Congenital Zika Program was created in early 2016 [6]. Approximately 10% of ZIKV-exposed cases in the continental US were within the referral region for the Congenital Zika Program at the height of the ZIKV epidemic [6].

Since 2016, a longitudinal cohort of children born in the US with in utero ZIKV exposure has been followed with neuroimaging and serial developmental assessments to monitor neurodevelopment over time. Some cases were referred during their mother’s pregnancy and had serial evaluations beginning in the womb [6], providing a unique opportunity to assess the long-term neurodevelopment of well-characterized children born in the US with in utero ZIKV exposure. The objective of this study was to determine if preschool-age children with antenatal ZIKV exposure born in the US have differences in neurodevelopment at preschool age compared to non-ZIKV-exposed children. The US children included in the cohort do not have Zika-related congenital disabilities or CZS. We hypothesized that ZIKV-exposed children may be more at risk of developmental delay than non-ZIKV-exposed controls.

## 2. Materials and Methods

### 2.1. Participants

We performed a prospective case–control study of children with in utero ZIKV exposure who were previously seen as a fetus or infants in the Congenital Zika Program at Children’s National Hospital [6] and non-ZIKV exposed controls in Washington, DC, United States (US). Eligible ZIKV-exposed children (participant cases) had clinical evaluations prenatally or after birth for concerns of antenatal ZIKV exposure between 2015–2017 [6]. Some eligible participants had fetal and early postnatal brain magnetic resonance imaging (MRI) and brain ultrasonographic imaging as part of a prospective cohort study [9,10] or as a clinical evaluation [6]. Mother–infant dyads were eligible for this study due to having ZIKV exposure determined by parental emigration from, or travel to, a country or US territory with circulating ZIKV during the ZIKV epidemic; maternal laboratory testing during pregnancy consistent with ZIKV infection performed within the correct sensitivity window, or ZIKV status unable to be ruled out by testing [6]; and child age of at least three years at the time of study enrollment. ZIKV laboratory testing was performed clinically. All eligible ZIKV-exposed children had either Confirmed ZIKV infection, which was defined as the pregnant parent having a positive ZIKV polymerase chain reaction (PCR) test and/or a positive Zika IgM serologic test with PRNT confirmation for Zika (>10) or Possible ZIKV infection with the pregnant parent’s testing either not performed or negative, but performed outside of sensitive testing windows, specifically PCR beyond 3 weeks of exposure or birth, and/or Zika IgM testing outside of the sensitive window of 2–12 weeks, post-exposure, respectively, per our prior publication [6]. All children were normocephalic at birth and did not have ZIKV-related congenital disabilities or a diagnosis of CZS [2]. Eligible ZIKV-exposed children from our clinical cohort were contacted to participate in this study by telephone, mail, email, text message, and WhatsApp.

Twelve non-ZIKV-exposed control children were prospectively enrolled. Eligible controls were 4- or 5-year-old children who did not meet any of the following exclusion criteria: inpatient hospitalization since birth for a chronic medical condition; major surgery with general anesthesia since birth; known congenital infection; failure to thrive; history of seizure; abnormal vision or hearing; developmental concerns expressed by caregiver; receipt of physical, occupational, or speech therapy; receipt of special education services in school; behavioral or psychological condition; planned delay in matriculation into primary school; or preterm birth (<36 weeks gestation); and metallic implant in body that is not compatible with MRI. Nine control participants were recruited through onsite clinics, a local farmers market, and research flyer distribution at local community spaces, including schools, daycares, libraries, grocery stores, and recreation centers, or referrals by friends. Three control children were recruited through a well-characterized healthy control cohort [11,12,13,14,15] or from a hospital database of children enrolled in the pediatric COVID-19 vaccine trials (Healthy Heroes Registry). Recruitment materials were presented in English and Spanish to attempt to recruit an equal number of controls to ZIKV-exposed cases and a similar distribution of primary languages among participants.

### 2.2. Ethics Approval

This study received approval from the Hospital’s Institutional Review Board. Parents provided written permission for their child’s study participation. Families were compensated for their time at each time point of participation. The study followed the Strengthening the Reporting of Observational Studies in Epidemiology (STROBE) reporting guideline. This study is registered on ClinicalTrials.gov, NCT04398901 (accessed on 29 August 2022).

### 2.3. Medical Record Review

Prenatal and child medical records for ZIKV-exposed cases were reviewed to ascertain the history of maternal ZIKV exposure, including whether it was due to travel or emigration and the country of exposure. Child medical records available in the electronic medical record were reviewed for all eligible ZIKV-exposed cases. Records were reviewed to ascertain the last known visit at the children’s hospital and any data available regarding neurodevelopmental outcomes, specialists seen, and the results of any fetal or postnatal neuroimaging. Available neuroimaging was reviewed by an experienced pediatric neuroradiologist. Participants’ race and ethnicity were also recorded from documentation in their medical records.

### 2.4. Caregiver-Completed Questionnaires and Child Observational Evaluations

ZIKV-exposed participants were evaluated using the ZIKV Outcome Toolbox (Table 1) at ages 3–4 and/or age 4–5 years between November 2020 and October 2022. Control participants completed one evaluation at age 4–5 years using the same ZIKV Outcome Toolbox. The study visits included caregiver-completed questionnaires and an in-person evaluation of multiple domains of child development. The ZIKV Outcome Toolbox was developed specifically for the evaluation of ZIKV-exposed children based on the breadth of developmental domains assessed. The team has also used the ZIKV Outcome Toolbox to evaluate child neurodevelopment following ZIKV exposure in the Atlántico Department, Colombia [16].

The toolbox includes questionnaires completed by caregivers and observational assessments of the child (Table 1) that can be administered in both English and Spanish languages. Standardized parent questionnaires included the Pediatric Evaluation of Disability Inventory (PEDI-CAT), which is a computer-based questionnaire that measures the functional domains of daily activities, mobility, social-cognitive, and responsibility (Pearson Q-global), and the Behavior Rating Inventory of Executive Function (BRIEF-P) which provides an early measure of child executive function (PAR, Inc., Lutz, FL, USA). Additional questionnaires completed by caregivers included information relating to the family, home environment, the child’s medical history, and caregivers’ occupation and education level. Mothers of children with ZIKV exposure were also asked to report feelings of stigmatization due to a diagnosis of ZIKV during their pregnancy. The Bracken School Readiness Assessment (BSRA) measures a child’s knowledge of five areas: colors, letters, numbers/counting, sizes/comparison, and shapes (Pearson Inc., New York, NY, USA). Child vocabulary and language development were assessed by either the Peabody Picture Vocabulary Test (PPVT) or the Spanish version, Test de Vocabulario en Imágenes Peabody (TVIP), based on the child’s preferred language (Pearson Inc., New York, NY, USA). Individual items differ between the PPVT and TVIP. Fine and gross motor skills were evaluated using the Movement Assessment Battery for Children-Second Edition (MABC-2) (Pearson Inc., New York, NY, USA). The MABC-2 evaluates three types of movement: manual dexterity, aiming and catching, and balance. Child weight, height, and head circumference were measured, and body mass index (BMI) was calculated. Due to the COVID-19 pandemic or relocation out of the region, some families agreed to participate only by completing online questionnaires and did not attend in-person study visits. The remaining children were evaluated in person. Three case participants were not able to complete and be scored for the in-person evaluations due to their diagnosis of autism spectrum disorder (ASD) or global developmental delay.

### 2.5. Statistical Analysis

The baseline characteristics of all participants were summarized using means and standard deviations for continuous variables and percentages for categorical variables. The analysis focused on the available 4-year-old evaluation data for both cases and controls. Scores for all outcomes were compared between cases and controls using the Mann–Whitney U Test. *p*-values were adjusted for multiple testing using the Benjamini-Hochberg false discovery rate (FDR) first for primary outcomes (BRIEF-P, MABC, and the Mobility domain score of PEDI-CAT) and then across all outcomes to evaluate secondary outcomes (BSRA, other scales of PEDI-CAT, and PPVT/TVIP). We used a 10% significance level for these adjusted p-values to maintain an FDR of ~10%. We explored the potential impact of confounding by comparing the estimate of the difference in the mean score of each outcome between cases and controls when the covariate was added to a regression model. We only explored covariates for which there were at least three subjects with outcome values in each group defined by the covariate and case/control status. This sensitivity analysis indicates that none of the covariates qualitatively changed the results (Table S1). As such, we report only the results based on the Mann–Whitney U test. We also used structural equation models (SEMs) to explore the overall difference in executive functioning based on the BRIEF-P. The BRIEF-P has five related but non-overlapping scales: Inhibit, Shift, Emotional Control, Working Memory, and Plan/Organize. Several studies have supported three latent factors that are derived from these five scales: Behavioral Regulation (Inhibit and Shift), Emotional Regulation (Shift and Emotional Control), and Cognitive Regulation (Working Memory and Plan/Organize) [17,18]. Sample sizes were too small to fit a single SEM in which the five scales define three factors representing different aspects of executive functioning. As such, two potential models were compared. The first uses the scoring based on previous studies to calculate the factor scores based on the five scales. These factor scores were then included in the model as being the result of a latent variable for executive functioning with a regression coefficient for whether a subject had ZIKV exposure predicting the executive functioning score. The second model used the five scales as measures of the latent variable for executive functioning, again with a regression coefficient for whether a subject had ZIKV exposure predictive of the executive functioning score. Both Akaike and Schwarz Bayesian information criteria (AIC and BIC) supported the three-factor scores as a better model. All analyses were conducted using R version 4.1.2, and the package lavaan() (version 4.3.0) was used to fit SEMs [19,20].

## 3. Results

### 3.1. Demographics

**Figure 1:**
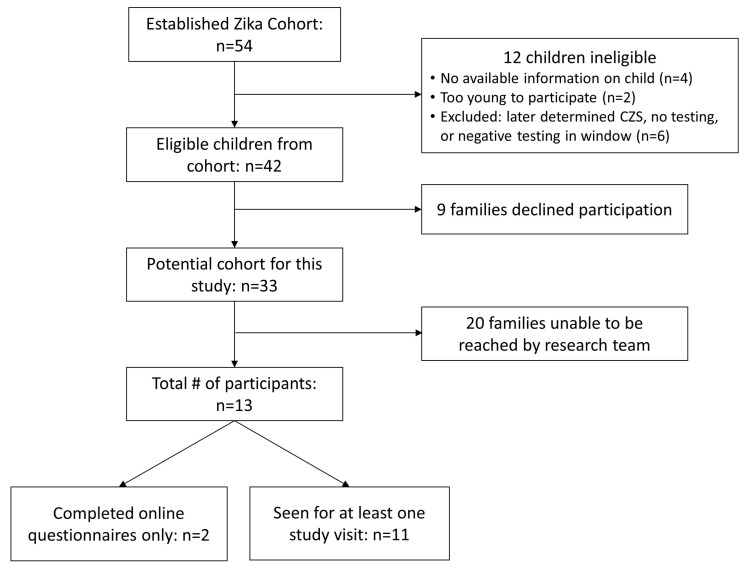
Recruitment of Zika-virus (ZIKV) exposed children. Thirteen ZIKV-exposed children were included in this study; 11 of the children had at least one in-person study visit at Children’s National Hospital, and 2 participated online only.

Forty-two mother–child dyads in the clinical ZIKV cohort met study inclusion criteria based on antenatal ZIKV exposure (Figure 1). Nine eligible mothers (21%) declined study participation, and 20 (48%) were not able to be contacted despite multiple attempts and methods of contact over two years. Thirteen (31%) mother–child dyads were enrolled, of whom 11 completed at least one in-person child evaluation. In nine (69%) ZIKV-exposed children, the parent had confirmed ZIKV infection during pregnancy, and in four (31%) children, parental ZIKV infection during pregnancy was considered possible since it was unable to be ruled out by laboratory testing (Table S2). Two families completed questionnaires over email due to relocation from the area. There was no difference in the percentage of participants with exposure by travel, as compared to emigration from an endemic area, between the 13 participants that consented to the study (54%), the 20 that were unable to be contacted (45%), and the 9 that declined (67%) (Fisher exact *p* = 0.565). Of the nine children who declined study participation, one child has an ASD diagnosis and accompanying severe language delays, as determined by medical record review.

The demographic, socioeconomic, and health characteristics of the 13 ZIKV-exposed children and 12 non-exposed controls are reported in Table 2. The age of the children at the evaluations was similar between the ZIKV-exposed children (mean = 4.9, SD = 0.3 years) and the non-exposed controls (mean = 4.8, SD = 0.4 years). Ethnicity was similar between ZIKV-exposed children and control participants. Parents’ educational attainment was different between ZIKV-exposed children and controls, with 38% of cases’ fathers and 73% of controls’ fathers receiving tertiary education, and 69% of cases’ mothers and 92% of controls’ mothers receiving the same. We did not collect data on other indicators of socioeconomic status, such as family income level.

Three mothers of ZIKV-exposed children (23%) reported feeling different from other mothers due to having ZIKV exposure during pregnancy, and all three reported having anxiety about the diagnosis (Table 2). Two of these three mothers have a child with either ASD or global developmental delay. While none of the mothers of the ZIKV-exposed cases reported worrying about their child’s health, four (30.8%) children were receiving rehabilitative therapy, three (23.1%) had behavior problems, and four (30.8%) had mood problems.

### 3.2. Neurodevelopmental Diagnoses and Neuroimaging in ZIKV-Exposed Cases

Ten of thirteen ZIKV-exposed children (77%) had neuroimaging performed during the fetal period, postnatal period, or both (Table 3). Four children had abnormal findings on the brain MRI performed between the neonatal period and the age of four years. In three of them, there was a destructive or encephaloclastic lesion, which is abnormal and is felt to likely relate to antenatal ZIKV exposure [9,10]. Three of the ZIKV-exposed children have a clinical diagnosis of a neurodevelopmental disorder: two have ASD (Case 12 and 13, Table 3), and one child has a global developmental delay (GDD) and is non-verbal, non-ambulatory, and has low–normal head size, but does not have the phenotype of CZS (Case 11, Table 3) and had a negative extensive genetic evaluation including whole exome sequencing. These three children with a diagnosis of a neurodevelopmental disorder comprised three of the four children in the cohort receiving therapy, three of the four with mood problems, and two of three with behavior problems. All three are male. Of note, one of the children with ASD has a family history of this diagnosis. The children had also been seen clinically as infants by a pediatric infectious disease specialist, and there was no concern for a different congenital infectious exposure [6].

### 3.3. Neurodevelopmental Performance of ZIKV-Exposed Cases and Non-Exposed Controls

The neurodevelopmental performance of the children who completed the evaluations is reported in Table 4. Three ZIKV-exposed children with ASD or global developmental delay (GDD) are described but did not receive a score for the in-person observational evaluations as they were unable to complete the assessments due to their neurodevelopmental disabilities.

**Figure 2:**
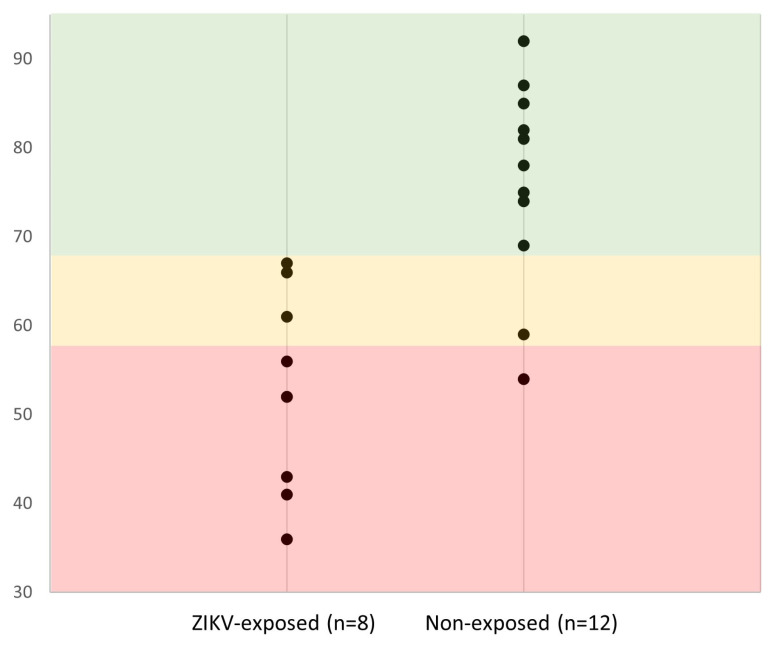
Movement ABC (MABC) standard scores, organized into “red”, “amber”, and “green” zones. Per the MABC manual (Pearson Inc., New York, NY, USA), a score in the “red zone” reflects a standard score of less than 57; the “amber zone” is between 57 and 67 inclusive; and the “green zone” is above 67. No ZIKV-exposed children scored within the green zone, compared to 9 out of 12 controls.

Child performance on the PPVT and TVIP did not show statistically significant differences between cases and controls, although the sample size for each group was small. Controls scored better for all three MABC domains, as well as BSRA categories. All unadjusted p-values for MABC domains and those for the BSRA shapes, sizes, comparisons, and composite domains on BSRA were less than 0.05. For MABC, five cases (61.1%; 95% CI: 29.5–88.1%) and two controls (19.2%; 95% CI: 3.6–43.6%) were in the “red zone” (standard score [SS] < 57), three cases (38.9%; 95% CI: 11.9–70.5%) and one control (11.5%; 95% CI: 0.9–32.8%) were in the “amber zone” (SS between 57 and 67 inclusive) and zero cases (5.6%; 95% CI: 0.00–26.2%) and nine controls (73.1%; 95% CI: 47.1–92.4%) were in the “green zone” (SS > 67) (Fisher exact FDR *p* = 0.02) (Figure 2). One case included in this analysis had a clinically significant score (t-score < 30) for PEDI-CAT (responsibility domain). No controls had a clinical elevation in any domain of the PEDI-CAT. While mean BRIEF-P t-scores were higher in ZIKV-exposed children than in controls for all scales and factors, consistent with higher case executive dysfunction, none of the *p*-values unadjusted for multiple testing were less than 0.05. Of the five scales, the inhibit score showed the largest estimated difference (8 t-score units, 95% CI: −2, 17), while the shift score showed the smallest estimated difference (~2 t-score units, 95% CI: −6, 7). The differences in the scales resulted in relatively large estimated differences for the behavioral and cognitive regulation factors (differences of 7.2 [95% CI: −4, 15] and 6.7 t-score units [95% CI: −5, 21], respectively). There was no difference between the number of cases and controls in clinically elevated domains for any BRIEF category (t-score > 65).

### 3.4. Neurodevelopmental Performance of Three Cases with Autism Spectrum Disorder (ASD) or Global Developmental Delay (GDD)

**Figure 3:**
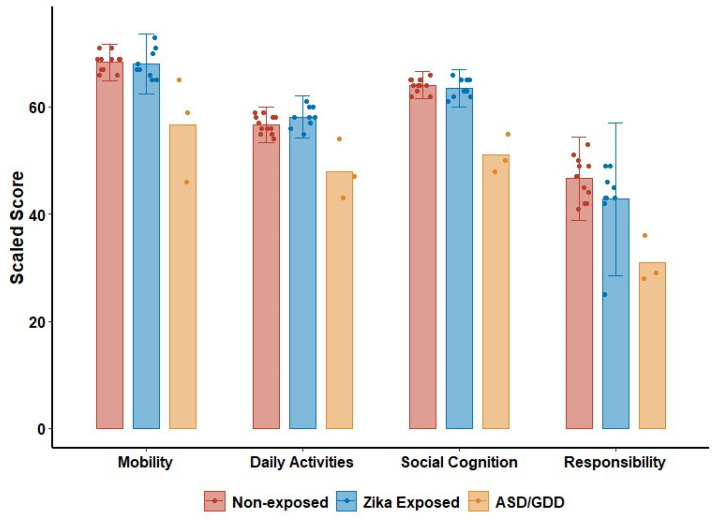
PEDI-CAT t-scores in non-exposed controls, ZIKV-exposed children without ASD/GDD, and ZIKV-exposed children with ASD/GDD. Scores on the PEDI-CAT Mobility, Daily Activities, Social Cognition, and Responsibility domains for ZIKV-exposed children with and without ASD (autism spectrum disorder) or GDD (global developmental delay) and non-exposed controls.

**Figure 4:**
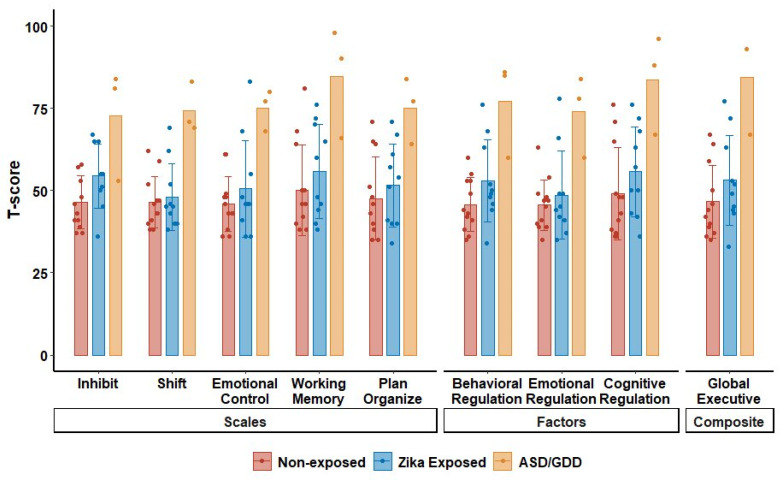
BRIEF-P t-score in non-exposed controls, ZIKV-exposed children without ASD/GDD, and ZIKV-exposed children with ASD/GDD. Scale, factor, and composite scores on the BRIEF-P for ZIKV-exposed children with and without ASD (autism spectrum disorder) or GDD (global developmental delay) and non-exposed controls.

Scores for three ZIKV-exposed children with a diagnosis of ASD or global developmental delay were excluded from the results in Table 4. All three children had t-scores in the clinically significant range for PEDI-CAT in all categories (T-score < 30 = moderate to severe disability) (Figure 3). All three children also scored in the clinically significant range for all categories of BRIEF-P, with mean t-scores above 65 for each category (Figure 4). Only one of the three children was able to attempt the assessments. While the child’s scores for MABC and TVIP fell in the lowest standard score (SS) categorization for each (SS = 1 and 55, respectively), the child was successfully able to identify 53% of school readiness concepts on BSRA (raw score = 45).

## 4. Discussion

This study finds a range of neurodevelopmental outcomes within a small cohort of children aged 4–5 years in the US with antenatal ZIKV exposure. Most ZIKV-exposed children are showing overall favorable developmental progress, but multi-domain evaluations suggest possible differences in motor skills, mood/behavior, areas of executive function, and school readiness that indicate a need for continued longitudinal follow-up. In addition, three children of the cohort have a diagnosis of ASD or global developmental delay. Along with a cohort of cases and controls from the Atlántico Department, Colombia, this US cohort study, although small, is among the longest-term outcome studies assessing child neurodevelopmental outcomes associated with congenital ZIKV exposure to date [21]. With the recency of the ZIKV epidemic and its broad implications for child development worldwide, it is crucial to understand how children with in utero ZIKV exposure continue to develop as they age in childhood. This study reflects insights into development for preschool-aged children born in the United States who were exposed to ZIKV prenatally due to parental travel to, or emigration from, an endemic country or region, and while they do not have Zika-related congenital disabilities or microcephaly have a range of neurodevelopmental outcomes.

This study utilized a combination of parental questionnaires and child evaluations, collectively called the ZIKV Outcome Toolbox (Table 1). Studies have shown that parents are accurate in providing information on their child’s current developmental status if questionnaires are criterion-specific and describe common activities that parents are able to observe [22,23,24]. Parental reports of executive function and functional domains of daily activities, mobility, social cognition, and responsibility were similar between cases and controls. The parental questionnaire components of the ZIKV Outcome Toolbox were selected for parents to be able to report areas of child development they observe at home. Areas of child development were also assessed by in-person evaluations of receptive vocabulary, motor development, and school readiness. We found some differences between cases and controls on direct child performance for fine and gross motor skills and a trend towards a difference in preschool readiness. In both groups, language development was generally similar. Thus, for all assessments except PEDI-CAT and TVIP, there was a trend toward better performance for controls. Of note, the three cases with ASD or global developmental delay did not contribute to the trends seen, since they were not able to complete those parts of the evaluation. It is unknown whether the trends seen are the result of true differences in case and control performance due to ZIKV exposure or were affected by the low power of the study, the presence of brain findings in some cases, or a result of parental education differences between cases and controls. Nevertheless, these childhood outcomes add to our understanding of the impacts of antenatal ZIKV exposure in the young child.

A notable finding in our study was that ZIKV-exposed children scored in the “red” (SS < 57) or “amber” (SS between 57 and 67) zones of the MABC more frequently than the healthy controls, indicating a potential difference in gross and fine motor coordination abilities between the ZIKV-exposed children and controls. In a previous study among young ZIKV-exposed children without CZS in Colombia, there was a significant difference in parent-reported child mobility between cases and controls as measured by the PEDI-CAT [16]. While our current study of children in the US did not find differences in PEDI-CAT mobility scores between cases and controls, the difference in MABC scores suggests that differences in movement abilities may be a potential long-term sequela of antenatal ZIKV exposure. Of note, some of the children in the ZIKV-exposed cohort have brain MRI abnormalities that may be related to ZIKV infection, although these findings are non-specific to the Zika virus but may be a reason for reduced motor performance. Future follow-up is needed to assess the trajectory of exposed children’s motor development and abilities.

Three of thirteen ZIKV-exposed children (23%) in this small cohort have a diagnosis of ASD or global developmental delay, in addition to one eligible child that was ZIKV exposed and declined enrollment (4 of 42 [9.5%] eligible ZIKV-exposed cases). It is unknown if their specific neurological outcome is associated with their exposure to ZIKV in utero, related to other factors, including genetic risk, or possibly multifactorial. For two of the four children with ASD or global developmental delay (one included in the study and one eligible child who did not enroll), there was a positive family history for ASD. This may indicate genetic susceptibility to neurodevelopmental impairment from infection or inflammatory exposure in utero, increasing potential risk in certain individuals and contributing to phenotypic variability [25]. The three children included in our cohort with these neurodevelopmental outcomes are male. Large population studies have shown that more males are affected by ASD and that factors such as gene-environment interactions and antenatal exposure to infection or inflammation may increase risk and underlying vulnerability [26]. A recent report from the Puerto Rico Autism Registry and the US Zika Pregnancy and Infant Registry (USZPIR) indicates the possibility of an increased risk for autism following antenatal ZIKV exposure, which requires further study and supports screening and neurodevelopmental assessments for all children with antenatal ZIKV exposure [27]. It is unknown if children unable to be contacted for our study (Figure 1) and who did not have follow-up records in our hospital medical records for review have similar differences in their neurodevelopment. However, among 55 children that our research team has followed in the Department of Atlántico, Colombia, with in utero ZIKV exposure, none have a diagnosis of ASD [16]. In a Brazilian cohort of children without microcephaly that had in utero ZIKV exposure, three had ASD at the age of 2 years [7]. Maternal infection with ZIKV during pregnancy may thus be a risk factor for ASD in offspring; however, making this association will require assessment of large cohorts.

Much of the reported early child outcome data following antenatal ZIKV exposure are from non-US birth cohorts, including a longitudinal cohort of children in Colombia followed by our research team [5,6,9,16]. However, the US Zika Pregnancy and Infant Registry has made important contributions to our understanding of the ZIKV burden in the United States. This is a national surveillance system that began during the ZIKV epidemic to monitor pregnancies with laboratory evidence of ZIKV infection and the outcomes of offspring from those pregnancies in the US states and territories. One hundred and twenty-two infants (5.4%) of 2248 infants reported to the US registry had a ZIKV-related congenital disability [28]. While children with ZIKV-related congenital disabilities had a greater frequency of neurologic sequelae and neurodevelopmental delays, those without ZIKV-related congenital disabilities still had risk. From this surveillance study, 129 of 1739 (7.4%) children without ZIKV-related congenital disabilities had a confirmed or possible developmental delay [28]. In a multi-specialty health brigade to the US Virgin Islands in 2021, children born to mothers with laboratory evidence of ZIKV infection during pregnancy received recommended ophthalmological, audiological, neurological, and neurodevelopmental screenings [29]. During the health brigade, 5 of 33 (15%) children were identified as needing subsequent specialty neurology care, 10 children (30%) were referred for speech therapy, and 4 (12%) children were referred for physical therapy [29].

As discussed, our research team has concurrently followed a cohort of 55 ZIKV-exposed children and 70 non-exposed controls in the Department of Atlántico, Colombia [16]. In both cohorts, BRIEF scores were higher in ZIKV-exposed cases than controls, although differences were only significant in the Colombian cohort, likely due to the small sample size of the US cohort. The BRIEF is a parent-completed questionnaire that assesses executive function, which is an area of cognitive development vulnerable to effects from early-in-life insults to the developing brain. There were also differences in the MABC scores between the ZIKV-exposed children in Colombia and those in the US; however, there are important factors to consider in interpreting these results. Twenty-eight of thirty (94%) children in Colombia who did the MABC scored in the green zone, while none of eight ZIKV-exposed children in the US scored in the green zone. In Colombia, the cases were a little younger, and not all children completed the MABC, so the number with scores in amber and red is likely not fully known. In the US cohort, the MABC findings indicate lower motor abilities in the eight children. It is possible that parental concern about their child’s motor development may have been a reason for choosing to participate in the study. While the MABC scores in Colombia did not reflect lower motor scores in ZIKV-exposed children compared to controls, there was lower mobility reported by the case parents in Colombia on the PEDI-CAT [16]. This may reflect lower adaptive mobility in Colombian children. Higher parental reports of mood problems or behavioral difficulties are also consistent between both the US and Colombia cohorts. The findings in both cohorts indicate that continued follow-up should include evaluation of cognitive, executive function, emotional regulation, and mobility domains [16].

The ZIKV Outcome Toolbox (originally described in [16]) was considered a major success of the study as it can be completed in under one hour, assesses a comprehensive variety of developmental domains, is easy to administer, and includes activities that child participants enjoy. As children in each cohort had primary languages of both English and Spanish, the activities in the ZIKV Outcome Toolbox were able to be adapted for the language of the participant (i.e., alternative uses of PPVT and TVIP). The research team responded to COVID-19-related challenges by administering some parent questionnaires online to shorten in-person child evaluations. Participant engagement was further maintained by providing individualized feedback to each participant’s caregiver. It is believed that strong participant engagement will have positive impacts on participant retention for future longitudinal follow-up, which is underway.

This study has several limitations, including challenges related to the timing of the COVID-19 pandemic, which may have limited recruitment and affected sample size. It is possible that caregivers of a child with ZIKV exposure during pregnancy who had a neurodevelopmental concern for their child were more likely to agree to participate than parents without concerns. A few eligible ZIKV-exposed cases declined participation in the study due to COVID-19 concerns and a reluctance to attend a study visit in a hospital setting, which in part reduced the potential participant population. Further, many ZIKV-exposed patients who were assessed during and after pregnancy were immigrants from ZIKV-endemic regions, including Latin America; it is noted (and presumed for those families who were unable to be contacted) that some potential participants moved back to their home countries or internally relocated and were thus unable to be enrolled in the study. It is unclear whether the demographics of those who relocated or were unable to be contacted embody shared characteristics that bias the study analysis. Four of the ZIKV-exposed children had parental testing in the category of ZIKV possible, so for them, it is unknown whether they were truly ZIKV-exposed. This is a limitation experienced by many clinically evaluated ZIKV cohorts due to short sensitivity windows for testing [6]. Another limitation is that three ZIKV-exposed children did not have neuroimaging during the perinatal period, so it is possible that they may have unrecognized structural brain abnormalities, although none had microcephaly. The study plans to obtain non-sedated brain MRIs in children when they are older.

Another challenge of this study was the enrollment of controls based on similar demographic characteristics (e.g., primary language) as ZIKV-exposed cases. Various methods of control recruitment were utilized, including community-based flyer distribution and recruitment through hospital clinics and research participant networks. Two children were referred to the study by a friend who had either participated in the study or seen a flyer. Despite the small sample size, through these methods, we achieved similar ethnic diversity between the ZIKV-exposed children and non-exposed control participants, which was important given the number of Hispanic participants in our case group. Enrollment of a greater number of controls would have been preferable for this case–control study but was not possible during the COVID-19 pandemic despite significant efforts. We did not record a detailed perinatal travel history for control participant parents; thus, it is possible, but unlikely, that a control could have had asymptomatic ZIKV exposure. While we did not collect data on the exact reason for study participation, it was noted that some parents of control participants were employees in health and research-related fields and had a general familiarity with the process of pediatric research participation. Future longitudinal follow-up will seek to understand reasons why parents of controls chose to participate and how those reasons may influence child performance. In addition, while there was higher educational attainment in control parents than in case parents, too few dyads had parental education less than community college to enable analyses of the impact of this covariate on the result.

## 5. Conclusions

In utero ZIKV exposure may present risks for neurodevelopmental delays in childhood, including effects on coordination, executive function, conceptual development, and regulatory behaviors. Differences in neurodevelopment may relate to structural brain changes, exposure to an abnormal intrauterine environment and maternal inflammation, and gene-environment interactions, among other factors [9,16,25]. Sample sizes would need to be larger to have reasonable power to be able to detect clinically meaningful changes in all outcomes. Since child neurodevelopment occurs over many years and the ability to assess many different domains of cognitive and neuromotor development improves with child age, it is essential to study older ages of children following antenatal or early-in-life exposures. Our findings may be generalizable to other prenatal infectious exposures and ZIKV-exposed children born outside of the United States. Furthermore, ASD risk may be increased for some children with antenatal ZIKV exposure, highlighting the importance of screening. Continued follow-up is needed to understand long-term impacts on cognitive development, motor coordination, literacy, numeracy, executive function, mood, and social skills of this cohort of US-born ZIKV-exposed children and controls, which is planned through age seven.

## Figures and Tables

**Table 1:** ZIKV Outcome Toolbox.

ZIKV Outcome Toolbox Item	Areas Assessed	Sub-Domains	Time (min)	Special Features
**Socioeconomic and Medical History** (parent-completed questionnaires)	Cohort characteristics: Medical health and home environment	General Health, Medical Conditions, Surgeries and Hospitalizations, Therapies, Psychiatric and Behavior History, Stigmatization of Zika Diagnosis, Housing and Home Environment, Family Occupation, Childcare, Parent Education, Family Transportation and Safety	10	Forms available in English or Spanish; completed on paper or as an online REDCap survey
**BRIEF-P** (parent-completed questionnaire)	Executive function ^a^	Inhibit, Shift, Emotional Control, Working Memory, Plan/ Organize, Inhibitory Self-Control Index, Flexibility Index, Emergent Metacognition Index, Global Executive Composite	10–15	English or Spanish forms are available; they can be completed online prior to the study visit
**MABC-2** (in-person child assessment)	Fine and gross motor ^a^	Manual Dexterity, Aiming and Catching, Balance, Overall Score	20	Can be administered in any language
**PEDI-CAT** (parent-completed questionnaire)	Functional developmental delay/disability ^a,b^	Mobility ^a^ (Primary Outcome), Daily Activities, Social-Cognitive, Responsibility	10–15	English or Spanish online tests are available; they can be completed before the study visit
**BSRA-3** (in-person child assessment)	School readiness ^b^	Colors, Letters, Numbers/Counting, Sizes/Comparisons, Shapes, Receptive School Readiness Composite	10–15	English or Spanish test forms available
**PPVT/TVIP** (in-person child assessment)	Receptive vocabulary ^b^	N/A	10–25	PPVT–English; TVIP–Spanish

^a^ Primary outcomes assessed include Executive function (BRIEF-P) and mobility (MABC-2 and PEDI-Cat Mobility). ^b^ Secondary outcomes assessed include School readiness (BSRA), vocabulary (PPVT or TVIP), and daily functional activities (other PEDI-CAT domains). Abbreviations: BRIEF-P: Behavior Rating Inventory of Executive Function–Preschool; BSRA-3: Bracken School Readiness Assessment–Third Edition; PEDI-CAT: Pediatric Evaluation of Disability Inventory Computer Adaptive Test; MABC-2: Movement Assessment Battery for Children–Second Edition; PPVT: Pearson Picture Vocabulary Test; TVIP: Test de Vocabulario en Imágenes Peabody.

**Table 2:** Sociodemographic characteristics of ZIKV-exposed children and non-exposed controls.

Characteristic	ZIKV-Exposed Children (N = 13)	Non-Exposed Controls (N = 12)
Male sex (n [%])	10 (76.9%)	5 (41.7%)
Years of age at visit (mean [SD])	4.9 (0.3)	4.8 (0.4)
Hispanic/Latino (n [%])	6 (46.2%)	4 (33.3%)
Race		
Asian	0 (0%)	1 (8.3%)
Black or African American	5 (38.5%)	3 (25.0%)
Caucasian	1 (7.7%)	2 (16.7%)
Other	6 (46.2%)	5 (41.7%)
Unknown/Not Reported	1 (7.1%)	1 (8.3%)
Exposed during travel to ZIKV endemic country	7 (53.8%)	-
Exposed before emigration from ZIKV endemic country	6 (46.2%)	-
Countries of origin or exposure	Dominican Republic, Mexico, El Salvador, Guatemala, St. Maarten, Cameroon, Thailand, Bahamas	-
Body mass index (mean [SD]) ^a^	17.6 (3.3)	15.6 (1.3)
Head Circumference Percentile (mean [SD]) ^a^	78.5 (29.6)	63.6 (24.8)
Education-level—mother (n [%])		
Primary School	0 (0%)	0 (0%)
Secondary School	2 (15.4%)	0 (0%)
High School	2 (15.4%)	1 (8.3%)
Technical School	3 (23.1%)	2 (16.7%)
University	6 (46.2%)	9 (75.0%)
NR	0 (0%)	0 (0%)
Education-level—father (n [%])		
Primary School	3 (23.1%)	0 (0%)
Secondary School	1 (7.7%)	0 (0%)
High School	3 (23.1%)	1 (8.3%)
Technical school	1 (7.7%)	2 (16.7%)
University	4 (30.8%)	6 (50.0%)
NR	0 (0%)	1 (8.3%)
None	1 (7.7%)	2 (16.7%)
Principal sustainer (n [%])		
Mother	4 (30.8%)	9 (75.0%)
Father	7 (53.8%)	2 (16.7%)
Both	2 (15.4%)	0 (0%)
Other or No Response	0 (0%)	1 (8.3%)
Number of children in the home (median) [Min, Max]	2 [1,3]	2 [1,4]
The participant was the oldest child in the home (n [%])	5 (38.5%)	7 (58.3%)
Mothers’ feelings about ZIKV diagnosis		
Feeling different than other mothers	3 (23.1%)	-
Embarrassment	1 (7.7%)	-
Sadness	1 (7.7%)	-
Anxiety	3 (23.1%)	-
Depression	0 (0%)	-
Decreased self-esteem	1 (7.7%)	-
Isolation	0 (0%)	-
Change in friendships	0 (0%)	-
Change in community feeling	0 (0%)	-
Change in partner/spouse relationship	0 (0%)	-
Child medical history (n [%])		-
Parent worried about child’s health	0 (0%)	0 (0%)
Vision problem	1 (7.7%)	2 (16.7%)
Hearing problem	1 (7.7%)	0 (0%)
Growth problem	0 (0%)	0 (0%)
Received therapy	4 (30.8%)	0 (0%)
Behavior problems	3 (23.1%)	0 (0%)
Mood problems	4 (30.8%)	0 (0%)

^a^ Excludes two ZIKV-exposed cases who did not complete an in-person study visit.

**Table 3:** Fetal and postnatal neuroimaging in ZIKV-exposed children.

Case #	Fetal Imaging	Fetal Imaging Results	Postnatal Imaging	Postnatal Imaging Results
1	MRI	Normal	Brain MRI	MRI (age 16 days): wedge-shaped cystic encephalomalacia in the right anterior parietal region.
	US		Head US	US: small choroid plexus cysts
2	MRI	MRI: Normal	None	---
	US	US: Choroid plexus cysts		
3	None	---	Head US	US: Normal
4	None	---	None	---
5	None	---	Brain CT	CT (age 43 days): Normal
6	MRI	MRI: Normal	Head US	US: small choroid plexus cysts
	US	US: Normal		
7	None	---	None	---
8	None	---	None	---
9	None	---	Head US	US: Normal
10	MRI	MRI: Multifocal deep white matter injury, corpus callosum thinner than expected; large bilateral germinolytic cysts.	Brain MRI	MRI (age 12 days): Multifocal deep white matter gliosis/necrosis, microhemorrhages, cerebral white matter volume loss, lateral ventricular germinolytic cysts, dysmorphology of temporal horns
	US	US: Mild bilateral ventriculomegaly, prominent lenticulostriate vessels		
11 *	None	---	Brain MRI	MRI (neonate): normal; follow-up MRI at the age of 4 years showed a mildly smaller than age-expected corpus callosum
12 *	None	---	Brain MRI	Normal
13 *	MRI	Normal	Brain MRI	MRI (age, 4 years): middle cranial fossa arachnoid cysts, mild multifocal cerebral white matter injury (gliosis), mild lateral ventriculomegaly
	US		Head US	US: mild left ventriculomegaly, mega cisterna magna

Postnatal imaging is performed during the neonatal period unless otherwise noted. An asterisk (*) indicates that this child has ASD/GDD and was excluded from some analyses. Abbreviations: MRI—magnetic resonance imaging; US—ultrasound; ZIKV—Zika virus. See References [9,10] for details on neuroimaging in Case 1.

**Table 4:** Neurodevelopmental performance in ZIKV-exposed children and non-exposed controls.

	ZIKV-Exposed (N = 10) ^a^	Non-Exposed (N = 12)	*p*-Value, FDR ^b^
**BRIEF-P Raw Score: Mean (SD) (*n_zv_* = 10) ^c^**			
Inhibit	54.4 (9.7)	46.4 (8.0)	0.295
Shift	48.0 (10.2)	46.3 (7.80)	0.8946
Emotional Control	50.5 (14.8)	46.0 (8.30)	0.805
Working Memory	55.9 (14.3)	50.1 (13.8)	0.524
Plan and organize	51.6 (12.6)	47.6 (12.7)	0.6258
Inhibitory Self-Control Index (ISCI)	53.0 (12.5)	45.8 (8.10)	0.3677
Flexibility Index (FI)	48.6 (13.4)	45.6 (7.67)	0.8523
Emergent Metacognition Index (EMI)	55.7 (13.7)	49.0 (14.1)	0.3677
General Executive Composite (GEC)	53.1 (13.7)	46.6 (11.1)	0.3677
**Movement ABC Standard Score (SS): Mean (SD) (*n_zv_* = 8)**			
Manual Dexterity	16.3 (6.32)	23.8 (8.26)	0.1387
Aiming and catching	14.9 (3.83)	19.3 (3.92)	0.1387
Balance	22.8 (5.75)	30.1 (5.47)	0.1387
Overall score	53.9 (11.1)	73.3 (14.6)	0.1085
**MABC Overall score categories: Number (proportion, 95% confidence interval) (*n_zv_* = 8)**			
“Red Zone” (SS < 57)	5 (61.1%; 29.5–88.1%)	2 (19.2%; 3.6–43.6%)	**0.02**
“Amber Zone” (57 ≤ SS ≤ 67)	3 (38.9%; 11.9–70.5%)	1 (11.5%; 0.9–32.8%)	
“Green Zone” (SS > 67)	0 (5.6%; 0.0–26.2%)	9 (73.1%; 47.1–92.4%)	
**PEDI-CAT Scaled Score: Mean (SD) (*n_zv_* = 9)**			
Daily Activities	58.1 (1.96)	56.8 (1.66)	0.2858
Mobility	68.0 (2.78)	68.3 (1.72)	0.7484
Social/Cognitive	63.6 (1.74)	64.1 (1.24)	0.6862
Responsibility	42.8 (7.16)	46.7 (3.89)	0.3359
**PPVT raw score: Mean (SD) ^d^**	89.3 (9.00)	109 (17.8)	0.171
**TVIP raw score: Mean (SD) ^d^**	115 (4.24)	115 (15.9)	0.8219
**BSRA % mastery: Mean (SD) (*n_zv_* = 8)**			
Colors	92.5 (17.5)	98.3 (5.77)	0.4752
Letters	47.4 (34.6)	73.3 (32.5)	0.171
Numbers	46.0 (31.8)	70.8 (29.0)	0.171
Sizes and Comparisons	55.1 (11.1)	70.8 (13.4)	0.1289
Shapes	55.6 (18.0)	74.6 (19.2)	0.1337
Total	52.4 (13.3)	75.3 (17.3)	0.1289

^a^ All results in Table 4 exclude three case participants with a diagnosis of ASD or GDD. ^b^ *p*-values adjusted for comparisons using the false discovery rate (FDR). ^c^ Online-only participants did not complete in-person tests, and some did not complete certain caregiver questionnaires. Case sample size for individual assessments indicated as (*n_zv_ =* [*n* ZIKV-exposed]). The sample size for non-exposed participants was 12 unless otherwise indicated. ^d^ The PPVT (Peabody Picture Vocabulary Test) was administered to English-speaking participants, and the TVIP (Test de Vocabulary in Imágenes Peabody) was administered to Spanish-speaking participants. Each test includes different items, scoring rules, and norms. Sample sizes (n = [n non-exposed, n ZIKV-exposed]) were (9, 4) for PPVT and (3, 2) for TVIP. **Abbreviations: BRIEF-P:** Behavior Rating Inventory of Executive Function—Preschool; **BSRA-3:** Bracken School Readiness Assessment—Third Edition; **PEDI-CAT:** Pediatric Evaluation of Disability Inventory Computer Adaptive Test; **MABC-2:** Movement Assessment Battery for Children—Second Edition; **PPVT:** Pearson Picture Vocabulary Test; **TVIP:** Test de Vocabulario en Imágenes Peabody; **SD:** standard deviation.

## Data Availability

The datasets generated during and/or analyzed during the current study are available from the corresponding author upon reasonable request.

## Supplementary Materials

The following supporting information can be downloaded at https://www.mdpi.com/article/10.3390/pathogens13070542/s1: Supplemental Table S1. Outcomes and Covariates. Supplemental Table S2. Maternal ZIKV testing results and exposure category for the ZIKV-exposed children.
