# Supplementary material for: Neurodevelopmental Outcomes of Preschoolers with Antenatal Zika Virus Exposure Born in the United States
Source: Pathogens. 2024 Jun 27;13(7):542. doi: 10.3390/pathogens13070542 (PMC11279881; doi:10.3390/pathogens13070542)
Supplement: Supplementary file 1 [file pathogens-13-00542-s001.zip › pathogens-3034530-supplementary.pdf]

Supplemental Table S1: Outcomes and Covariates

		Difference Between Cases and Controls				Bonferroni Significance
Outcome	Covariate included in model*	Estimate	LCL	UCL	P-value	
BRIEF: Inhibit, t-score	None	7.983	0.122	15.844	0.0469	0.003571429
BRIEF: Inhibit, t-score	Age	7.723	-0.106	15.552	0.0529	
BRIEF: Inhibit, t-score	Gender	6.508	-1.551	14.568	0.1073	
BRIEF: Inhibit, t-score	Ethnicity	7.585	-0.547	15.718	0.0658	
BRIEF: Inhibit, t-score	Number of children in home, including participant	8.513	0.869	16.158	0.0309	
BRIEF: Inhibit, t-score	Home type	7.427	-1.079	15.933	0.0832	
BRIEF: Inhibit, t-score	Home ownership	6.505	-1.659	14.668	0.1114	
BRIEF: Inhibit, t-score	Family worry about paying bills?	8.472	-0.021	16.966	0.0505	
BRIEF: Shift, t-score	None	1.667	-6.314	9.647	0.6678	
BRIEF: Shift, t-score	Age	1.628	-6.596	9.852	0.6833	
BRIEF: Shift, t-score	Gender	-0.216	-8.166	7.734	0.9553	
BRIEF: Shift, t-score	Ethnicity	1.323	-6.955	9.600	0.7417	
BRIEF: Shift, t-score	Number of children in home, including participant	1.387	-6.734	9.508	0.7247	
BRIEF: Shift, t-score	Home type	1.189	-7.484	9.862	0.7766	
BRIEF: Shift, t-score	Home ownership	-1.288	-8.273	5.696	0.7029	
BRIEF: Shift, t-score	Family worry about paying bills?	1.222	-7.449	9.893	0.7705	
BRIEF: Emotional, t-score	None	4.500	-5.913	14.913	0.3781	
BRIEF: Emotional, t-score	Age	4.500	-6.239	15.239	0.3914	
BRIEF: Emotional, t-score	Gender	1.309	-8.473	11.091	0.7825	
BRIEF: Emotional, t-score	Ethnicity	4.065	-6.740	14.869	0.4408	
BRIEF: Emotional, t-score	Number of children in home, including participant	4.358	-6.382	15.099	0.4063	
BRIEF: Emotional, t-score	Home type	3.784	-7.458	15.026	0.4886	
BRIEF: Emotional, t-score	Home ownership	1.063	-7.579	9.705	0.7990	
BRIEF: Emotional, t-score	Family worry about paying bills?	5.000	-6.075	16.075	0.3555	
BRIEF: Memory, t-score	None	5.817	-6.729	18.362	0.3450	
BRIEF: Memory, t-score	Age	5.426	-7.121	17.974	0.3767	

BRIEF: Memory, t-score	Gender	3.993	-9.114	17.101	0.5313
BRIEF: Memory, t-score	Ethnicity	4.940	-7.923	17.804	0.4314
BRIEF: Memory, t-score	Number of children in home, including participant	6.419	-6.166	19.004	0.2991
BRIEF: Memory, t-score	Home type	3.859	-9.222	16.941	0.5431
BRIEF: Memory, t-score	Home ownership	2.703	-9.749	15.154	0.6538
BRIEF: Memory, t-score	Family worry about paying bills?	5.361	-8.311	19.033	0.4208
BRIEF: Plan, t-score	None	4.017	-7.297	15.331	0.4676
BRIEF: Plan, t-score	Age	3.719	-7.698	15.135	0.5036
BRIEF: Plan, t-score	Gender	1.086	-9.997	12.170	0.8396
BRIEF: Plan, t-score	Ethnicity	3.189	-8.391	14.768	0.5711
BRIEF: Plan, t-score	Number of children in home, including participant	4.619	-6.650	15.888	0.4017
BRIEF: Plan, t-score	Home type	2.222	-9.586	14.029	0.6973
BRIEF: Plan, t-score	Home ownership	1.315	-10.107	12.738	0.8116
BRIEF: Plan, t-score	Family worry about paying bills?	4.083	-8.131	16.297	0.4914
BRIEF: GEC, tscore	None	6.517	-4.516	17.550	0.2322
BRIEF: GEC, tscore	Age	6.245	-4.919	17.408	0.2562
BRIEF: GEC, tscore	Gender	3.841	-7.100	14.781	0.4715
BRIEF: GEC, tscore	Ethnicity	5.802	-5.541	17.144	0.2978
BRIEF: GEC, tscore	Number of children in home, including participant	6.892	-4.345	18.130	0.2147
BRIEF: GEC, tscore	Home type	5.114	-6.603	16.830	0.3713
BRIEF: GEC, tscore	Home ownership	3.135	-7.384	13.654	0.5391
BRIEF: GEC, tscore	Family worry about paying bills?	6.528	-5.477	18.533	0.2683
BRIEF: ISCI, t-score	None	7.250	-1.942	16.442	0.1155
BRIEF: ISCI, t-score	Age	7.091	-2.301	16.483	0.1305
BRIEF: ISCI, t-score	Gender	4.807	-4.149	13.764	0.2753
BRIEF: ISCI, t-score	Ethnicity	6.766	-2.735	16.267	0.1525
BRIEF: ISCI, t-score	Number of children in home, including participant	7.557	-1.811	16.925	0.1077
BRIEF: ISCI, t-score	Home type	6.514	-3.383	16.410	0.1837
BRIEF: ISCI, t-score	Home ownership	4.721	-3.903	13.345	0.2652
BRIEF: ISCI, t-score	Family worry about paying bills?	7.806	-2.035	17.646	0.1129

BRIEF: Flexibility, t-score	None	3.017	-6.497	12.531	0.5159
BRIEF: Flexibility, t-score	Age	2.991	-6.818	12.801	0.5309
BRIEF: Flexibility, t-score	Gender	0.199	-8.828	9.227	0.9636
BRIEF: Flexibility, t-score	Ethnicity	2.544	-7.302	12.389	0.5950
BRIEF: Flexibility, t-score	Number of children in home, including participant	2.779	-6.979	12.537	0.5581
BRIEF: Flexibility, t-score	Home type	2.330	-7.962	12.622	0.6401
BRIEF: Flexibility, t-score	Home ownership	-0.532	-8.443	7.380	0.8893
BRIEF: Flexibility, t-score	Family worry about paying bills?	2.972	-7.358	13.303	0.5531
BRIEF: EMI, t-score	None	6.700	-5.708	19.108	0.2734
BRIEF: EMI, t-score	Age	6.279	-6.057	18.615	0.3001
BRIEF: EMI, t-score	Gender	4.661	-8.202	17.525	0.4575
BRIEF: EMI, t-score	Ethnicity	5.619	-6.970	18.209	0.3619
BRIEF: EMI, t-score	Number of children in home, including participant	7.342	-5.044	19.727	0.2299
BRIEF: EMI, t-score	Home type	4.822	-8.183	17.826	0.4461
BRIEF: EMI, t-score	Home ownership	3.766	-8.761	16.293	0.5356
BRIEF: EMI, t-score	Family worry about paying bills?	6.556	-6.996	20.107	0.3229
MABC: dexterity score	None	-7.583	-14.834	-0.332	0.0413
MABC: dexterity score	Age	-7.653	-15.232	-0.075	0.0480
MABC: dexterity score	Gender	-7.835	-15.757	0.088	0.0523
MABC: dexterity score	Ethnicity	-7.739	-15.556	0.079	0.0521
MABC: dexterity score	Number of children in home, including participant	-7.516	-15.031	-0.001	0.0500
MABC: dexterity score	Home type	-7.100	-14.721	0.521	0.0658
MABC: dexterity score	Home ownership	-7.783	-15.420	-0.145	0.0463
MABC: dexterity score	Family worry about paying bills?	-7.196	-15.079	0.687	0.0709
MABC: Aiming and catching score	None	-4.458	-8.183	-0.734	0.0216
MABC: Aiming and catching score	Age	-4.733	-8.518	-0.949	0.0172
MABC: Aiming and catching score	Gender	-5.597	-9.264	-1.929	0.0050

MABC: Aiming and catching score	Ethnicity	-4.092	-8.056	-0.127	0.0438
MABC: Aiming and catching score	Number of children in home, including participant	-4.710	-8.312	-1.108	0.0134
MABC: Aiming and catching score	Home type	-4.630	-8.473	-0.787	0.0212
MABC: Aiming and catching score	Home ownership	-3.761	-7.282	-0.240	0.0377
MABC: Aiming and catching score	Family worry about paying bills?	-4.130	-8.258	-0.002	0.0499
MABC: Balance, overall score	None	-7.333	-12.684	-1.983	0.0100
MABC: Balance, overall score	Age	-7.773	-13.172	-2.375	0.0074
MABC: Balance, overall score	Gender	-7.189	-13.038	-1.340	0.0190
MABC: Balance, overall score	Ethnicity	-7.392	-13.164	-1.621	0.0151
MABC: Balance, overall score	Number of children in home, including participant	-7.090	-12.476	-1.705	0.0129
MABC: Balance, overall score	Home type	-6.530	-12.059	-1.001	0.0235
MABC: Balance, overall score	Home ownership	-6.978	-12.556	-1.401	0.0172
MABC: Balance, overall score	Family worry about paying bills?	-7.098	-12.898	-1.297	0.0196
MABC: Overall score	None	-19.375	-32.191	-6.559	0.0052
MABC: Overall score	Age	-20.160	-33.302	-7.018	0.0049
MABC: Overall score	Gender	-20.620	-34.505	-6.735	0.0061
MABC: Overall score	Ethnicity	-19.222	-33.046	-5.399	0.0093
MABC: Overall score	Number of children in home, including participant	-19.316	-32.612	-6.021	0.0070
MABC: Overall score	Home type	-18.260	-32.119	-4.401	0.0130
MABC: Overall score	Home ownership	-18.522	-31.880	-5.164	0.0094
MABC: Overall score	Family worry about paying bills?	-18.424	-32.259	-4.589	0.0122
PEDICAT: Mobility scaled score	None	-0.333	-2.393	1.727	0.7386
PEDICAT: Mobility scaled score	Age	-0.319	-2.433	1.795	0.7550
PEDICAT: Mobility scaled score	Gender	-0.037	-2.279	2.205	0.9725
PEDICAT: Mobility scaled score	Ethnicity	0.227	-1.558	2.013	0.7922
PEDICAT: Mobility scaled score	Number of children in home, including participant	-0.479	-2.576	1.619	0.6374

PEDICAT: Mobility scaled score	Home type	-0.254	-2.491	1.982	0.8133	
PEDICAT: Mobility scaled score	Home ownership	-0.220	-2.389	1.948	0.8328	
PEDICAT: Mobility scaled score	Family worry about paying bills?	-0.009	-2.071	2.053	0.9926	

* Only covariates for which the outcome had at least 3 observations in each group were included.

Supplemental Table S2: Maternal ZIKV testing results and exposure category for the ZIKV-exposed children

Case Participant #	ZIKV lab testing result	ZIKV exposure category *
1	Maternal ZIKV PCR positive	ZIKV infection
2	Maternal ZIKV IgM negative (tested outside of sensitivity window)	ZIKV possible
3	Maternal ZIKV PRNT positive	ZIKV infection
4	Maternal ZIKV IgM negative (tested outside of sensitivity window)	ZIKV possible
5	Maternal ZIKV PRNT positive	ZIKV infection
6	Maternal ZIKV PRNT positive	ZIKV infection
7	Maternal ZIKV PRNT positive	ZIKV infection
8	Maternal ZIKV PRNT positive	ZIKV infection
9	Maternal ZIKV PRNT positive	ZIKV infection
10	Maternal ZIKV IgM negative (tested outside of sensitivity window)	ZIKV possible
11	Maternal ZIKV PRNT positive	ZIKV infection
12	Maternal ZIKV PRNT positive	ZIKV infection
13	No maternal testing, symptomatic in 1st trimester following travel. Infant testing ZIKV PCR and IgM negative.	ZIKV possible

* The ZIKV exposure category is described in the methods section and in Reference 6.
